# Association Between Household Online Grocery Delivery Service Use and Food and Drink Purchase Behavior in England: Cross-Sectional Analysis

**DOI:** 10.2196/41540

**Published:** 2023-12-19

**Authors:** Amy Yau, Cherry Law, Laura Cornelsen, Jean Adams, Emma Boyland, Thomas Burgoine, Frank de Vocht, Martin White, Steven Cummins

**Affiliations:** 1 Department of Public Health, Environments and Society Faculty of Public Health and Policy London School of Hygiene & Tropical Medicine London United Kingdom; 2 Department of Agri-Food Economics & Marketing School of Agriculture, Policy and Development University of Reading Reading United Kingdom; 3 Medical Research Council (MRC) Epidemiology Unit University of Cambridge Cambridge United Kingdom; 4 Department of Psychology Institute of Population Health University of Liverpool Liverpool United Kingdom; 5 Population Health Sciences Bristol Medical School University of Bristol Bristol United Kingdom; 6 National Institute for Health Research Applied Research Collaboration West (NIHR ARC West) Bristol United Kingdom

**Keywords:** food and beverages, food preferences, supermarkets, internet, consumer behavior, lifestyle, diet, inequality, food purchase, sociodemographic factors, grocery purchase, online grocery, online purchase, public health, online, delivery, grocery, diet

## Abstract

**Background:**

Online grocery delivery services (OGDSs) are a popular way of acquiring food. However, it is unclear whether OGDS use is associated with the healthiness of purchases and whether there are sociodemographic differences in OGDS use. If so, the increased prevalence of OGDS use may have implications for population diet, and differential OGDS use could contribute to diet inequalities.

**Objective:**

This study aimed to examine whether OGDS use varies by sociodemographic characteristics and is associated with the amount and types of groceries purchased.

**Methods:**

Item-level take-home food and drink purchase data (n=3,233,920 items) from households in London and the North of England were available from the 2019 UK Kantar fast-moving consumer goods panel (N=1911). Purchases were categorized as being bought online or in-store. We used logistic regression to estimate the likelihood of an above-median frequency of OGDS use by sociodemographic characteristics. We used Poisson regression to estimate the differences in energy and nutrients purchased by households that had above- and below-median OGDS use and the proportion of energy purchased from products high in fat, salt, and sugar (HFSS) online versus in-store among households that used both shopping methods (n=665).

**Results:**

In total, 668 (35%) households used OGDSs at least once in 2019. Of the households that used OGDSs, the median use was 5 occasions in 2019. Households were more likely to have above-median use in London versus in the North of England (odds ratio 1.29, 95% CI 1.01-1.65) and if they had a higher annual household income (odds ratio 1.56, 95% CI 1.02-2.38 for ≥£50,000 [US $64,000] vs <£20,000 [$25,600]). Households with above-median OGDS use had a higher weekly mean purchase of energy by 1461 (95% CI 1448-1474) kcal per person compared with households with below-median OGDS use. For households that used a combination of in-store and online shopping, HFSS products made up a lower proportion (–10.1%, 95% CI –12% to –8.1%) of energy purchased online compared to in-store.

**Conclusions:**

Differences in grocery purchases between households with above- and below-median OGDS use could have positive or negative consequences. The extra energy purchased among households with above-median OGDS use could lead to overconsumption or food waste, which has negative consequences for population and environmental health. Alternatively, this extra energy may be replacing out-of-home purchasing, which tends to be less healthy, and may be beneficial for the population diet. Households made fewer HFSS purchases when shopping online compared to in-store, which may be due to differences in the shopping environment or experience, such as fewer promotions and advertisements when shopping online or not having to transport and carry purchases home. As higher-income households used OGDS more frequently, the implications of this sociodemographic pattern on dietary inequalities must be explored.

## Introduction

There has been an increasing interest in whether the use of digital platforms to purchase foods impacts on the type of products purchased and, therefore, population diet and health. Much of this research has focused on the purchase of takeaway foods [1-3], but fewer studies have explored the use of online services for grocery purchases. The use of online grocery delivery services (OGDS) is an increasingly popular method of purchasing food and drink products for preparation and consumption at home. Although online delivery services have been available for over 20 years, they have grown rapidly in the past decade, with recent consumer adoption and growth in the use of these services further accelerated by the COVID-19 pandemic [4,5]. These services enable groceries to be ordered online for home delivery or to be picked up through “click and collect,” where purchased products are selected and packed in-store ready for collection by the consumer. The industry predicts further growth, with around half of those who used OGDS for the first time during the pandemic intending to continue [6].

OGDS may plausibly have positive and negative impacts on dietary behaviors [7-10]. Positive impacts might arise through changes in the shopping experience compared to traditional in-store purchasing. For example, products are presented symbolically in online channels rather than physically in stores, which may decrease their vividness and reduce consumers’ desire to purchase [11]. OGDS also increase the lag time between product choice and food acquisition [12]. It is theorized that people make more healthful food choices when the outcome (ie, acquisition or consumption of food) is further into the future [9], as there is a decreased focus on immediate gratification [11,12]. OGDS may also improve access to food, thereby potentially increasing access in communities with poor availability or quality of local food shopping opportunities or enabling those with poor access to transport to take advantage of home delivery [13-15]. Almost all households in Great Britain are covered by at least 1 supermarket delivery service, with 3 of the major supermarkets each providing coverage to over 98% of households [15].

There may also be negative effects on dietary behaviors. Sizing of products may be more difficult to determine online, leading to over- or underpurchasing. One UK study found that there were differences in the size of products, fewer price promotions, and less front-of-pack labeling for products available online when compared to similar products in-store [16]. Online delivery may also be more appealing for larger, heavier items and increase bulk buying (leading to overconsumption and waste), while consumers may be less inclined to purchase healthier perishable goods such as fresh fruit and vegetables [10].

Who accesses these services and what they purchase online may be influenced by sociodemographic characteristics, which could also plausibly influence dietary inequalities [8]. For example, many OGDSs have minimum spend requirements and delivery fees, meaning that more affluent households may have greater access to and use of these services compared to more disadvantaged households [10]. OGDS use may also be affected by household size, composition, age, and other sociodemographic characteristics. Similar differences have been observed for online delivery services for takeaway foods, where the use of these services was more likely among adults who were younger, male, from an ethnic minority background, highly educated, or had children [1]. Such differences may have implications for dietary inequalities if OGDS use influences purchasing behavior [7,8,11].

Whether OGDS affects the dietary quality of purchases has not been well studied. Some limited evidence from the United States suggests that the share of consumer expenditure on healthy products is higher when grocery purchases are made online compared to in-store [9,11,12,17]. From intervention studies that use online delivery services to improve diet and nutrition, including in low-income populations, there is some evidence that they can improve the healthiness of purchases [18,19].

Overall, the evidence on who uses OGDS and how the use of these services may have either positive or negative effects on dietary behaviors is limited. A deeper understanding of the ways that OGDSs are associated with purchasing behavior could help inform future policies and interventions aiming to improve population diet and reduce dietary inequalities. This study, using large-scale consumer panel data from England, aims to generate evidence in this area by (1) exploring the sociodemographic correlates of OGDS use and (2) exploring whether the use of OGDS is associated with overall grocery food and drink purchases and purchases of unhealthier products that are high in fat, salt, and sugar (HFSS).

## Methods

### Study Design

We used data on household take-home grocery food and drink purchasing from the 2019 Kantar fast-moving consumer goods (FMCG) panel. FMCG are products sold quickly and at a relatively low cost, including food and drink. Kantar, a commercial consumer data company, continually recruits around 30,000 households in the United Kingdom to a live panel via email and post using quota sampling. The households largely reflect the key sociodemographic characteristics of the geographical region they have been sampled from. The data available for this analysis were from a study examining the association between outdoor HFSS food and drink advertising restrictions in London and household food and drink purchases [20]. Households in our analysis were randomly selected from Kantar households in London or the North of England (North West, North East, and Yorkshire and the Humber regions). The included households (N=1911) recorded individual food and drink purchases (n=3,233,799 items) from December 31, 2018, to December 29, 2019. Our findings are reported in accordance with the Strengthening the Reporting of Observational Studies in Epidemiology (STROBE) guidelines (Multimedia Appendix 1).

### Ethical Considerations

Ethical approval was not required for the analysis of anonymized secondary data. Upon joining, participants agreed to the terms and conditions of the Kantar FMCG panel, which state that their data may be used for research purposes. Panelists receive around £100 (~US $128) worth of vouchers for their participation per year.

### Purchases and Nutrient Data

Participating households recorded all food and drink items purchased and brought into the home using a handheld barcode scanner. Nonbarcoded products, such as loose fruits and vegetables, were recorded using bespoke barcodes. Kantar provided nutrient data based on direct assessment of product nutrient labels in outlets twice a year or using product nutrient label images provided by Brandbank, an FMCG product database. Where this nutritional information was not available, Kantar obtained nutritional values from similar products or an average value for the product type was used.

We used nutrient data provided by Kantar to assess the healthiness of products by categorizing purchases as HFSS or non-HFSS, based on the UK’s nutrient profiling model (NPM), which was developed by the Food Standards Agency in 2004/2005 [21]. We chose this classification as it has been used in policies and regulations in the United Kingdom previously [22,23]. An NPM score for each product was calculated by adding points for energy, sugar, sodium, and saturated fat and subtracting points for protein, fiber, fruit, nut, and vegetable content. Information on the energy, sugar, sodium, saturated fat, protein, and fiber content of each purchase was provided by Kantar. Kantar categorizes product markets as high, mixed (medium) or low in fruit, nuts, and vegetables, which we then used to assign individual products with 5 (>80%), 1 (>40% and ≤80%), or 0 (≤40%) points for fruit, nut, and vegetable content. The higher the final NPM score, the less healthy the product. We used the recommended cut-offs of ≥4 for foods and ≥1 for drinks to classify a product as HFSS. We additionally split purchases into 35 food groups based on (1) product market and submarket classifications provided by Kantar and (2) whether products were healthier (non-HFSS) or less healthy (HFSS). These food groups have been used in other studies and are described in Table S1 in Multimedia Appendix 1 [24].

### Sociodemographic Characteristics

Sociodemographic data are collected by Kantar annually. We had access to data on sex, age (years), and occupational social class of the main food shopper, number of adults and children in the household, and household income. Occupational social class was based on the National Readership Survey social grade [25]. We categorized occupational social class as high (AB—higher and intermediate managerial, administrative, and professional occupations), middle (C1C2—supervisory, clerical and junior managerial, administrative, and professional occupations), and low (DE—semiskilled and unskilled manual occupations and unemployed, including retired). Gross annual household income bands were available for 1608 (84.1%) households (<£20,000, £20,000-£29,999, £30,000-£39,999, £40,000-£49,999, and ≥£50,000 [A currency exchange rate of £1=US $1.28 is applicable.]). Height and weight were self-reported by 1544 (80.8%) of the main food shoppers and used to calculate BMI (kg/m^2^).

### Outcomes

#### Mode of Purchase

Panelists reported the type of store each purchase was from, including whether purchases were made through the internet. Purchases were determined to be online or in-store purchases according to this information. Click and collect services were included in OGDS, as store type information was based on where purchases were made rather than how they were delivered.

#### Purchases

Household purchases, stratified by online or in-store, were summed over the 1-year study period. We then divided this by household size and the 52 study weeks to obtain means per person per week for outcomes: energy (kcal), fat (g), saturated fat (g), sugar (g), salt (g), items (packs), and expenditure (£). To account for energy purchased, we also looked at mean weekly outcomes per 100 kcal purchased for fat (g/100 kcal), saturated fat (g/100 kcal), sugar (g/100 kcal), salt (g/100 kcal), and expenditure (£/100 kcal). We further explored the proportion of purchases that were HFSS and the proportion of energy purchased from HFSS products and from each of the 35 food groups.

### Statistical Analyses

All items purchased on the same day using the same mode of purchase (online or in-store) were considered as having been purchased in the same shopping occasion. The use of OGDS was highly skewed (Figure S1 in Multimedia Appendix 1). Most households did not use OGDS in 2019, and many households that did use them did so infrequently. Among households that used OGDS, the median use was 5 occasions in 2019. We explored differences in purchases between households that used OGDS above and below the median as we theorized that households with very low OGDS use (eg, 1-2 occasions in a year) would not be representative of typical users.

Using a logistic regression model, we examined the association between sociodemographic characteristics (sex, age group, region, occupational social class, household income, number of adults in the household, and presence of children in the household) and OGDS use (above- or below-median). Using Poisson regression models, we explored the association between OGDS use and total purchases of energy, nutrients (overall and per 100 kcal), items, and expenditure (overall and per 100 kcal). Marginal effects were obtained, and pairwise comparison was used to estimate the difference in mean purchases per person per week. We also examined the association between OGDS use and the proportion of purchases that were HFSS using linear regression models. For households that used both online and in-store purchasing methods (n=665), we compared the mean proportion of energy purchased from HFSS products and the 35 food groups through the 2 shopping methods. To examine the associations between OGDS use and purchases independent of household sociodemographic characteristics, all models were adjusted for sex, age, and occupational social class of the main food shopper, region, household income, and proportion of household members that were children (number of children divided by household size). The logistic regression model used to investigate the association between sociodemographic characteristics and OGDS use was also mutually adjusted for other sociodemographic characteristics. All analyses were conducted in Stata SE 16 (Stata Corp).

### Sensitivity Analyses

As the cut-offs for categorizing households by the frequency of OGDS could have been made in several ways, we also explore the differences between regular users (which we defined as households using OGDS ≥26 occasions in 2019) and nonregular users (<26 occasions) to see if our results were sensitive to changes in cut-off. As only 147 households were regular OGDS users, we did not use this as our main analysis due to reduced statistical power. We also ran the analyses comparing households that had any OGDS use (n=668) with those that had no OGDS use (n=1243). As mentioned above, we did not use this as our main analysis as we theorized that households with very low OGDS use would not be representative of typical users. Using a subsample of households where the main food shopper’s BMI was known (n=1544), we also reran our analyses, additionally adjusting for BMI—a potential confounder of the relationship between OGDS use and purchasing behavior—to see if this changed our findings. BMI was categorized into 2 groups: not overweight (<25 kg/m^2^) and overweight (≥25 kg/m^2^).

## Results

### Study Population and OGDS Use

In total, 1911 households were included in our analyses (Table 1). Most households had a female main food shopper (n=1391, 72.8%), were in the middle social class group (n=1133, 59.3%), had 2 adults (n=1062, 55.6%), and had no children (n=1358, 71.1%). Households varied in their household income and age of the main shopper. Of the 1911 included households, 668 (35%) used OGDS at least once in 2019. Almost all households that used OGDS also shopped in-store (n=665, 99.6%). Of households that used OGDS, the median use was 5 (IQR 1-22) occasions in 2019. When stratified by above- (≥5 occasions) and below- (<5 occasions) median OGDS use, there were 353 (18.5%) households above and 1558 (81.5%) below the median including households that did not use OGDS at all. Without adjustment, female main shoppers, younger main shoppers, households in London, households with higher annual income, and households with children were more likely to have above-median OGDS use.

**Table 1:** Sociodemographic characteristics of the study sample.

		Total users (N=1911), n (%)	Above-median OGDS^a^ users (n=353), n (%)	Below-median OGDS users (n=1558), n (%)	Chi-square (*df*)		*P* value	
**Sex of the main shopper**								.05
	Male	520 (27.2)	81 (23)	439 (28.2)	3.98 (1)			
	Female	1391 (72.8)	272 (77.1)	1119 (71.8)				
**Age of the main shopper (years)**								<.001
	18-34	244 (12.8)	49 (13.9)	195 (12.5)	31.84 (4)			
	35-44	349 (18.3)	82 (23.2)	267 (17.1)				
	45-54	501 (26.2)	117 (33.1)	384 (24.7)				
	55-64	436 (22.8)	60 (17)	376 (24.1)				
	≥65	381 (19.9)	45 (12.8)	336 (21.6)				
**Region**						.02		
	London	939 (49.1)	194 (55)	745 (47.8)	5.87 (1)			
	North of England	972 (50.9)	159 (45)	813 (52.2)				
**Occupational social class**								.14
	Low	334 (17.5)	64 (18.1)	270 (17.3)	4.00 (2)			
	Middle	1133 (59.3)	194 (55)	939 (60.3)				
	High	444 (23.2)	95 (26.9)	349 (22.4)				
**Household income^b^**								<.001
	<£20,000	388 (20.3)	65 (18.4)	323 (20.7)	43.11 (5)			
	£20,000-£29,999	323 (16.9)	49 (13.9)	274 (17.6)				
	£30,000-£39,999	267 (14)	35 (9.9)	232 (14.9)				
	£40,000-£49,999	217 (11.4)	57 (16.2)	160 (10.3)				
	≥£50,000	413 (21.6)	110 (31.2)	303 (19.5)				
	Unknown	303 (15.9)	37 (10.5)	266 (17.1)				
**Adults in the household**								.14
	1	424 (22.2)	67 (19)	357 (22.9)	3.98 (2)			
	2	1062 (55.6)	212 (60.1)	850 (54.6)				
	≥3	423 (22.2)	74 (21)	351 (22.5)				
**Children in the household**								.001
	Yes	553 (28.9)	128 (36.3)	425 (27.3)	11.29 (1)			
	No	1358 (71.1)	225 (63.7)	1133 (72.7)				
**BMI (kg/m^2^)**								.11
	Not overweight	601 (31.5)	101 (28.6)	500 (32.1)	4.34 (2)			
	Overweight	943 (49.4)	171 (48.4)	772 (49.6)				
	Unknown	367 (19.2)	81 (23)	286 (18.4)				

^a^OGDS: online grocery delivery service.

^b^A currency exchange rate of £1=US $1.28 is applicable.

### Food and Drink Purchases

Overall, households purchased a median of 9808 (IQR 6988-13,375) kcal and spent £21.40 (IQR £14.20-£31.40) per person per week (Table 2). Most food and drink items were purchased in-store, with 2,891,843 (89.4%) items purchased in-store and 341,956 (10.6%) items purchased online. HFSS purchases accounted for 1,231,882 (38.1%) of total items purchased and households purchased a median of 4.8 (IQR 3.2-6.9) HFSS items per person per week. Without adjustment, households with above-median OGDS use had higher purchases of energy and salt, purchased more items and spent more compared to households with below-median OGDS use. Households with above-median OGDS use also purchased marginally more salt per 100 kcal, spent more per 100 kcal purchased, and purchased marginally less sugar per 100 kcal compared to households with below-median OGDS use in our unadjusted analysis.

**Table 2:** Unadjusted sample purchases of energy, nutrients, items, and expenditure per person per week for households with above- and below-median online grocery delivery service (OGDS) use.

	Total users (N=1911), median (IQR)	Above-median OGDS users (n=353), median (IQR)	Below-median OGDS users (n=1558), median (IQR)	Chi-square (*df*=1)	*P* value
Energy (kcal)	9808 (6988-13,375)	10,220 (7302-13,801)	9728 (6836-13,332)	3.83	.05
Fat (g)	412.9 (290.9-574.8)	434.3 (306.3-598.0)	408.5 (288.5-571.9)	1.87	.17
Saturated fat (g)	155.0 (105.7-225.8)	161.1 (113.2-230.4)	153.2 (103.9-225.0)	2.20	.14
Sugar (g)	433.9 (294.8-623.6)	437.5 (307.9-623.2)	433.8 (290.5-623.6)	0.00	.94
Salt (g)	31.1 (21.4-44.1)	33.3 (23.8-45.9)	30.5 (21.1-43.3)	4.80	.03
Items (n)	14.7 (10.3-20.5)	16.0 (11.6-21.8)	14.5 (10.1-20.3)	7.21	.01
HFSS^a^ items (n)	4.8 (3.2-6.9)	5.0 (3.5-7.1)	4.7 (3.1-6.8)	3.46	.06
Expenditure (£)^b^	21.4 (14.2-31.4)	24.2 (16.9-35.3)	20.6 (13.7-30.6)	18.61	<.001
Fat per 100 kcal	4.25 (3.90-4.60)	4.25 (3.91-4.59)	4.25 (3.90-4.61)	0.03	.87
Saturated fat per 100 kcal	1.62 (1.43-1.81)	1.64 (1.44-1.85)	1.62 (1.43-1.80)	0.18	.67
Sugar per 100 kcal	4.49 (3.83-5.18)	4.36 (3.77-5.11)	4.52 (3.85-5.20)	4.71	.03
Salt per 100 kcal	0.32 (0.27-0.37)	0.32 (0.28-0.37)	0.31 (0.27-0.37)	3.83	.05
Expenditure per 100 kcal	0.22 (0.18-0.27)	0.24 (0.20-0.29)	0.21 (0.17-0.26)	37.00	<.001

^a^HFSS: high in fat, salt, and sugar.

^b^A currency exchange rate of £1=US $1.28 is applicable.

### Sociodemographic Correlates of OGDS Use

In our adjusted models, households where the main shopper was female were marginally more likely than those with male shoppers to have above-median OGDS use (odds ratio [OR] 1.38, 95% CI 1.04 to 1.83; Table 3). London households were more likely to have above-median OGDS use than households in the North of England (OR 1.29, 95% CI 1.01-1.65). Households in the highest 2 income groups (£40,000-£49,999 and ≥£50,000) were more likely to have above-median OGDS use than households with an income <£20,000 (OR 1.70, 95% CI 1.09-2.66 and OR 1.56, 95% CI 1.02-2.38, respectively). No difference was observed by age group of main shopper, occupational social class, number of adults, or the presence of children in the household.

**Table 3:** Sociodemographic differences in online grocery delivery service (OGDS) use (N=1911) using a logistic regression model adjusted for sex and age of the main food shopper, occupational social class, household income, the proportion of household members that were children, and region.

		Above-median OGDS use		
		Odds ratio (95% CI)		Percentage, mean (95% CI)
**Sex of the main shopper**				
	Male	Reference	15.3 (12.2-18.3)	
	Female	*1.38* (*1.04*-*1.83*)^a^	19.7 (17.6-21.8)	
**Age of the main shopper (years)**				
	18-34	Reference	18.9 (13.9-23.8)	
	35-44	1.15 (0.77-1.74)	21.1 (16.6-25.6)	
	45-54	1.28 (0.86-1.90)	22.8 (19.1-26.5)	
	55-64	0.75 (0.48-1.18)	14.9 (11.3-18.5)	
	≥65	0.65 (0.40-1.06)	13.3 (9.5-17.0)	
**Region**				
	North of England	Reference	16.7 (14.3-19.0)	
	London	*1.29* (*1.01*-*1.65*)	20.3 (17.7-22.9)	
**Occupational social class**				
	Low	Reference	22.4 (17.5-27.3)	
	Middle	0.72 (0.51-1.01)	17.4 (15.2-19.6)	
	High	0.78 (0.52-1.18)	18.6 (15.0-22.2)	
**Household income^b^**				
	<£20,000	Reference	17.5 (13.3-21.7)	
	£20,000-£29,999	0.88 (0.58-1.34)	15.7 (11.7-19.8)	
	£30,000-£39,999	0.72 (0.45-1.15)	13.3 (9.2-17.4)	
	£40,000-£49,999	*1.70* (*1.09*-*2.66*)	26.3 (20.4-32.1)	
	≥£50,000	*1.56* (*1.02*-*2.38*)	24.7 (20.1-29.2)	
	Unknown	0.66 (0.42-1.05)	12.4 (8.7-16.2)	
**Adults in household**				
	1	Reference	18.1 (14.0-22.2)	
	2	1.12 (0.80-1.57)	19.8 (17.4-22.2)	
	≥3	0.84 (0.56-1.25)	15.7 (12.4-19.1)	
**Children in household**				
	No	Reference	18.1 (15.9-20.3)	
	Yes	1.08 (0.80-1.46)	19.2 (15.8-22.7)	

^a^Italic formatting indicates significance at 95% CI.

^b^A currency exchange rate of £1=US $1.28 is applicable.

### Association Between OGDS Use and Total Food and Drink Purchases

After adjustment for sociodemographic and household characteristics, households with above-median OGDS use had higher mean purchases of energy (1461 kcal, 95% CI 1448-1474 kcal), fat (64.8 g, 95% CI 62.1-67.6 g), saturated fat (28.5 g, 95% CI 26.8-30.2 g), sugar (63.9 g, 95% CI 61.1-66.7 g), salt (6.1 g, 95% CI 5.4-6.9 g), and items (3 items, 95% CI 2.5-3.5 items) per person per week (Table 4). These households also spent more on groceries per person per week (mean £6.30, 95% CI £5.70-£7.00) than those with below-median OGDS use. However, there was no difference in nutrients purchased per 100 kcal between the 2 groups. When food products and drink products were separated, food accounted for most of the differences observed, although the significant associations were the same for both food and drink (Table S2 in Multimedia Appendix 1).

**Table 4:** Incidence rate ratio (95% CI) and predicted difference in adjusted weekly mean (95% CI) purchases of energy, nutrients, items per person, and expenditure on groceries per person between households with above- and below-median online grocery delivery service (OGDS) use using a Poisson model adjusted for sex and age of the main food shopper, occupational social class, household income, the proportion of household members that were children, and region.

	Incidence rate ratio (95% CI)	Predicted outcome for above-median OGDS use (n=353), mean (95% CI)	Predicted outcome for below-median OGDS use (n=1558), mean (95% CI)	Predicted difference, mean (95% CI)
Energy (kcal)	1.14 (1.14 to 1.14)	11,927 (11,916 to 11,939)	10,466 (10,461 to 10,471)	*1461* (*1448* to *1474*)^a^
Fat (g)	1.14 (1.14 to 1.15)	512.3 (509.8 to 514.8)	447.4 (446.4 to 448.5)	*64.8* (*62.1* to *67.6*)
Saturated fat (g)	1.17 (1.16 to 1.18)	200.4 (198.8 to 201.9)	171.9 (171.2 to 172.5)	*28.5* (*26.8* to *30.2*)
Sugar (g)	1.13 (1.13 to 1.14)	544.6 (542.0 to 547.2)	480.7 (479.6 to 481.8)	*63.9* (*61.1* to *66.7*)
Salt (g)	1.18 (1.16 to 1.20)	40.3 (39.6 to 41.0)	34.2 (33.9 to 34.5)	*6.1* (*5.4* to *6.9*)
Items (n)	1.19 (1.16 to 1.22)	18.8 (18.4 to 19.3)	15.8 (15.6 to 16.0)	*3.0* (*2.5* to *3.5*)
Expenditure (£)^b^	1.27 (1.24 to 1.30)	29.9 (29.3 to 30.5)	23.6 (23.4 to 23.9)	*6.3* (*5.7* to *7.0*)
Fat per 100 kcal	1.00 (0.94 to 1.06)	4.3 (4.2 to 4.5)	4.3 (4.2 to 4.4)	–0.0 (–0.3 to 0.2)
Saturated fat per 100 kcal	1.02 (0.93 to 1.12)	1.7 (1.5 to 1.8)	1.6 (1.6 to 1.7)	0.0 (–0.1 to 0.2)
Sugar per 100 kcal	0.99 (0.93 to 1.04)	4.5 (4.3 to 4.8)	4.6 (4.5 to 4.7)	–0.1 (–0.3 to 0.2)
Salt per 100 kcal	1.03 (0.84 to 1.27)	0.3 (0.3 to 0.4)	0.3 (0.3 to 0.4)	0.0 (–0.1 to 0.1)
Expenditure per 100 kcal	1.13 (0.89 to 1.43)	0.3 (0.2 to 0.3)	0.2 (0.2 to 0.3)	0.0 (–0.0 to 0.1)

^a^Italic formatting indicates significance at 95% CI.

^b^A currency exchange rate of £1=US $1.28 is applicable.

### Association Between OGDS Use and Purchases of HFSS Products

There were no differences in HFSS purchases between households that had above- and below-median OGDS use in our adjusted models (Table 5). At a more disaggregated level, households with above-median OGDS use purchased more or the same amount as households that had below-median use across healthier and less healthy food groups (Table S3 in Multimedia Appendix 1).

**Table 5:** Coefficient (95% CI) and predicted difference in adjusted weekly mean (95% CI) energy; nutrients; items from high in fat, salt, and sugar purchases as a proportion of all grocery food and drink purchases; and expenditure on products high in fat, salt, and sugar as a proportion of all food and drink grocery expenditure between households with above- and below-median online grocery delivery service (OGDS) use using a linear regression model adjusted for sex and age of the main food shopper, occupational social class, household income, proportion of household members that were children, and region.

	Coefficient (95% CI)	Predicted outcome for above-median OGDS use (n=353), mean (95% CI)	Predicted outcome for below-median OGDS use (n=1558), mean (95% CI)	Predicted difference, mean (95% CI)
Energy	0.00 (–0.01 to 0.01)	52.7 (51.8 to 53.7)	52.5 (52.0 to 52.9)	0.2 (–0.8 to 1.3)
Fat	0.01 (–0.00 to 0.02)	71.3 (70.3 to 72.2)	70.6 (70.2 to 71.1)	0.6 (–0.4 to 1.6)
Saturated fat	0.01 (–0.00 to 0.02)	77.0 (76.1 to 77.9)	76.0 (75.5 to 76.4)	1.0 (–0.0 to 2.0)
Sugar	–0.00 (–0.02 to 0.01)	56.5 (55.1 to 57.9)	56.7 (56.0 to 57.3)	–0.2 (–1.8 to 1.4)
Salt	0.01 (–0.01 to 0.02)	64.4 (63.3 to 65.5)	63.7 (63.2 to 64.2)	0.7 (–0.5 to 1.9)
Items	–0.00 (–0.01 to 0.01)	37.6 (36.7 to 38.6)	37.7 (37.2 to 38.1)	–0.0 (–1.1 to 1.0)
Expenditure	–0.00 (–0.01 to 0.01)	39.5 (38.5 to 40.5)	39.7 (39.2 to 40.2)	–0.2 (–1.4 to 0.9)

### Association Between Mode of Shopping and Purchases Among OGDS Users

Among households that used both online and in-store shopping methods (n=665), the mean proportion of energy purchased from HFSS products was –10.1% (95% CI –12% to –8.1%) lower in online compared to in-store shopping occasions (43.3%, 95% CI 41.4%-45.2% vs 53.3%, 95% CI 52.5%-54.1%) in the adjusted model. When stratified by food group, online purchases had proportionately more energy from vegetables (1%, 95% CI 0.2%-1.8%), healthy nonmilk-based drinks (1.6%, 95% CI 0.7%-2.4%), and alcohol (1.2%, 95% CI 0.4%-2.1%) and proportionally less energy from some HFSS food groups, including puddings and biscuits (–3.3%, 95% CI –4.1% to –2.5%), chocolate and confectionery (–1.5%, 95% CI –2.2% to –0.7%), and savory snacks (–0.8, 95% CI –1.3% to –0.2%; Table S4 in Multimedia Appendix 1). Healthy nonmilk-based drinks were nonalcoholic and not milk-based (eg, milkshakes and coffee drinks). Examples of nonmilk-based drinks included carbonated drinks and juice drinks. Healthy refers to non-HFSS according to the UK nutrient profiling model. The proportion of energy was also lower from carbohydrate food groups (healthy bread: –3.1%, 95% CI –3.7% to –2.5%; less healthy bread: –0.7, 95% CI –0.8% to –0.5%; morning goods: –0.6%, 95% CI –0.8% to –0.4%), dairy products (reduced fat milk: –0.9, 95% CI –1.2% to –0.6%; high fat milk: –0.4, 95% CI –0.6% to –0.2%; less healthy cheese: –1.2, 95% CI –1.7% to –0.7%, healthy cheese: –0.01, 95% CI –0.02% to 0%), protein (–0.6%, 95% CI –1.1% to 0%), and ready meals (healthy: –1.2%, 95% CI –1.6% to –0.8%; less healthy: –0.7, 95% CI –1.2% to –0.2) for online shopping occasions.

### Sensitivity Analyses

The difference in total grocery purchases between regular OGDS users and nonregular users was larger compared to the main analysis (2208 kcal, 95% CI 2188-2228 kcal vs 1461 kcal, 95% CI 1448-1474 kcal; Table S5 in Multimedia Appendix 1). There were no qualitative differences in the proportion of HFSS purchases between regular OGDS users versus nonregular users and above- versus below-median users (Table S6 in Multimedia Appendix 1). The association between households with any versus no OGDS use and total purchases was in the same direction as the main analysis, but the magnitude of difference was smaller (756 kcal, 95% CI 746-766 kcal vs 1461 kcal, 95% CI 1448-1474 kcal; Table S7 in Multimedia Appendix 1). Although the point estimates for HFSS purchases were similar for any versus no OGDS use and above- versus below-median use, a marginally higher proportion of energy, fat, and saturated fat from HFSS products was observed among households that had any OGDS use compared to households with none (Table S8 in Multimedia Appendix 1). Adjusting for BMI did not qualitatively change the association between OGDS use and total food and drink purchases (Table S9 in Multimedia Appendix 1) or OGDS use and HFSS purchases (Table S10 in Multimedia Appendix 1).

## Discussion

### Summary of Main Findings

Overall, in our sample, 35% (n=668) of London and the North of England households used OGDS at least once, with OGDS users having a median use of 5 occasions in 2019. Almost all households that used OGDS also purchased foods in-store. Higher-income households, households with a female main food shopper, and households in London versus the North of England had a higher likelihood of above-median OGDS use. Households with above-median OGDS use had higher mean purchases of groceries per person per week, in terms of energy (1461 kcal), number of food and drink items (3 items), and expenditure (£6.30). Nutrients purchased per 100 kcal, expenditure on purchases per 100 kcal, and the proportion of total purchases that were HFSS were similar in both groups. However, households that used both shopping modes purchased a 10.1% (95% CI 8.1%-12%) lower proportion of energy from HFSS products from their online compared with in-store purchases.

### Interpretation of Findings

Almost no households in our sample exclusively used OGDS for their grocery shopping. OGDS use therefore appears to complement, rather than substitute, in-store grocery shopping. The extra energy purchased among households with above-median OGDS use, compared with below-median OGDS use, could lead to overconsumption or food waste, which has negative consequences for population health and environmental health. Alternatively, more energy purchased for in-home consumption could be because these households eat out less. If this is the case, additional grocery purchases may be beneficial for population diet as consuming out-of-home food is associated with weight gain and higher intakes of energy, fat, salt, and sugar [26,27].

No significant differences in the healthiness of purchasing between households according to their OGDS use were observed, with households with above-median OGDS use purchasing greater amounts of both HFSS and non-HFSS products. However, there was a difference in the healthiness of purchasing within households, with their online purchases having fewer HFSS products than their in-store purchases. This means that households made healthier choices online or preferentially purchased their healthier products online. As our analysis was a comparison within households, the differences observed here cannot be due to household characteristics and are likely due to factors that influence the shopping experience (eg, vividness of products, time lag between shopping and receiving items, exposure to promotions and front-of-pack labeling, preference for purchasing some items online or in-store, and ability to browse products online vs in-store) and, in turn, purchasing behavior [11,12,28]. Online shopping environments have been found to be more supportive of healthier diets through fewer advertisements and promotions and a lack of product placement to encourage HFSS purchases. A healthier food environment in an online setting, compared to a physical setting, is supported by studies from the United States, which found OGDS to be associated with purchasing healthier food [9,11,12,17]. The higher proportion of energy from fruit and vegetables in households’ online shops suggests that households were not deterred from purchasing perishable goods without being able to choose products personally, which was a concern highlighted in previous research [10]. Differences in the online food environment, compared to the physical food environment, may have implications for population health, especially as OGDS use prevalence increases. Therefore, further research is needed to understand why households choose OGDS and how it relates to in-store purchasing and out-of-home purchasing to give us a better understanding of total food purchases. With the COVID-19 pandemic having encouraged greater use of OGDS [4,5], future studies should examine short- and long-term changes in food purchasing behaviors and their associations with population diet.

While we have some understanding of, and are trying to regulate, aspects of the in-store food environment [29], less is known about how online platforms influence purchases and whether and how these should (or should not) be regulated. Some studies have shown positive results for interventions that manipulate the online food environment to encourage healthier purchasing [28,30,31]. The online food environment could be used to increase access to fresh food, improve access to nutritional information, and reduce exposure to HFSS advertising [10,19]. While there are fewer promotions online compared to in-store in the United Kingdom (24% of products vs 32% of products), front-of-pack labeling was only found for 42% of products online compared to 74% in-store [16]. Regulations could be helpful in harnessing the elements of OGDSs that may improve population health while limiting the elements that may have negative impacts.

### Strengths and Limitations

While online platforms for grocery purchasing have been available for many years, little is known about their association with food and drink purchasing behavior in the United Kingdom. Our findings contribute to the evidence base on OGDS use and food purchasing behavior. Purchase data are a good indicator of food and drink consumption [32,33]. However, they do not tell us about the intrahousehold distribution of purchases, which we assumed to be equal across all household members, including children. This, of course, will not have been the case and provides only an estimate, which likely underestimates purchased energy and nutrients per adult for households with children. Purchase data also do not tell us about food waste. Having data at the item level with the day on which purchases were made and whether this was online or in-store allowed us to establish OGDS use by number of occasions. However, it is unknown whether households may report online and in-store purchases differently. Households may also have forgotten to report purchases or chosen to not report less healthy purchases due to social desirability. The lower average weekly household grocery food expenditure in this analysis (£21.40) compared to the UK’s Living Costs and Food Survey (£32.12) may be explained by the inclusion of only 2 UK regions in our study and the unweighted nature of our data, which limits its accuracy in reflecting population-level averages [34]. We were able to compare online and in-store purchases for households that used both modes of shopping, which removed potential confounding from household characteristics. However, we were only able to assess purchases brought into the home and could not consider out-of-home purchases, so we were unable to capture overall household food purchasing. We were also unable to equivalize household income as we did not have information on the age of the children in the household. However, all models were adjusted for the proportion of household members that were children. Errors in the nutrient data, imputed values where data were missing, and the crude estimation of fruit, nut, and vegetable content may have affected the accuracy of our categorization of products as HFSS or non-HFSS. We were unable to explore how online shopping interacts with other forms of food acquisition.

### Conclusions

In this study, OGDS use was greater among higher-income and London households. Households that had above-median OGDS use purchased a mean of 1461 kcal more energy per person per week through their grocery purchases. The differences in grocery purchases between households with above- and below-median OGDS use could have positive or negative consequences. The extra energy purchased through groceries among households with above-median OGDS use could lead to overconsumption or food waste, a problem for population health and the environment. Alternatively, more energy purchased for in-home consumption could plausibly replace out-of-home consumption, which may be beneficial to population diet, as out-of-home food tends to be less healthy. Households purchased fewer HFSS purchases when shopping online compared to in-store, which may be due to differences in the shopping environment or experience, such as fewer promotions and advertisements when shopping online or not having to transport and carry purchases home. As higher-income households used OGDS more frequently, the implications of this sociodemographic patterning on dietary inequalities need to be explored. Further research is needed to investigate the relationship between OGDS use and the healthiness of purchasing in different population subgroups. With the online food environment becoming an increasingly important retail channel for household food purchasing, it should be an important consideration when designing food system policies and interventions that aim to improve population diet and reduce dietary inequalities.

## Appendix

Multimedia Appendix 1 Supplementary Files.

## Abbreviations

FMCG: fast-moving consumer good
HFSS: high in fat, salt, and sugar
NPM: nutrient profiling model
OGDS: online grocery delivery service
OR: odds ratio
STROBE: Strengthening the Reporting of Observational Studies in Epidemiology

## Data Availability

The data sets generated and analyzed during this study are not publicly available due to the terms of our data agreement with Kantar. Data are available to purchase by contacting Kantar directly.
