# Supplementary material for: Association Between Household Online Grocery Delivery Service Use and Food and Drink Purchase Behavior in England: Cross-Sectional Analysis
Source: JMIR Public Health Surveill. 2023 Dec 19;9:e41540. doi: 10.2196/41540 (PMC10762614; doi:10.2196/41540)
Supplement: Multimedia Appendix 1 [file publichealth_v9i1e41540_app1.docx]

**Text S1.** STROBE Statement—Checklist of items that should be included in reports of ***cross-sectional studies***

|  | Item No | Recommendation |
| --- | --- | --- |
| **Title and abstract** | 1 | (*a*) Indicate the study’s design with a commonly used term in the title or the abstract |
|  |  | (*b*) Provide in the abstract an informative and balanced summary of what was done and what was found |
| Introduction | | |
| Background/rationale | 2 | Explain the scientific background and rationale for the investigation being reported **Page 2** |
| Objectives | 3 | State specific objectives, including any prespecified hypotheses **Page 2** |
| Methods | | |
| Study design | 4 | Present key elements of study design early in the paper **Pages 2-3** |
| Setting | 5 | Describe the setting, locations, and relevant dates, including periods of recruitment, exposure, follow-up, and data collection **Pages 2-3** |
| Participants | 6 | (*a*) Give the eligibility criteria, and the sources and methods of selection of participants **Pages 2-3** |
| Variables | 7 | Clearly define all outcomes, exposures, predictors, potential confounders, and effect modifiers. Give diagnostic criteria, if applicable **Page 3** |
| Data sources/ measurement | 8* | For each variable of interest, give sources of data and details of methods of assessment (measurement). Describe comparability of assessment methods if there is more than one group **Pages 3-4** |
| Bias | 9 | Describe any efforts to address potential sources of bias **Page 10** |
| Study size | 10 | Explain how the study size was arrived at **Pages 2-3** |
| Quantitative variables | 11 | Explain how quantitative variables were handled in the analyses. If applicable, describe which groupings were chosen and why **Pages 3-4** |
| Statistical methods | 12 | (*a*) Describe all statistical methods, including those used to control for confounding **Pages 3-4** |
|  |  | (*b*) Describe any methods used to examine subgroups and interactions **N/A** |
|  |  | (*c*) Explain how missing data were addressed **Pages 3-4** |
|  |  | (*d*) If applicable, describe analytical methods taking account of sampling strategy **N/A** |
|  |  | (*e*) Describe any sensitivity analyses **Page 4** |
| Results | | |
| Participants | 13* | (a) Report numbers of individuals at each stage of study—eg numbers potentially eligible, examined for eligibility, confirmed eligible, included in the study, completing follow-up, and analysed **Page 4-5** |
|  |  | (b) Give reasons for non-participation at each stage **Page 4** |
|  |  | (c) Consider use of a flow diagram |
| Descriptive data | 14* | (a) Give characteristics of study participants (eg demographic, clinical, social) and information on exposures and potential confounders **Pages 4-6** |
|  |  | (b) Indicate number of participants with missing data for each variable of interest **Page 3** |
| Outcome data | 15* | Report numbers of outcome events or summary measures **Pages 5-6** |
| Main results | 16 | (*a*) Give unadjusted estimates and, if applicable, confounder-adjusted estimates and their precision (eg, 95% confidence interval). Make clear which confounders were adjusted for and why they were included **Pages 4-9** |
|  |  | (*b*) Report category boundaries when continuous variables were categorized **Pages 3-4** |
|  |  | (*c*) If relevant, consider translating estimates of relative risk into absolute risk for a meaningful time period |
| Other analyses | 17 | Report other analyses done—eg analyses of subgroups and interactions, and sensitivity analyses **Page 9** |
| Discussion | | |
| Key results | 18 | Summarise key results with reference to study objectives **Page 9** |
| Limitations | 19 | Discuss limitations of the study, taking into account sources of potential bias or imprecision. Discuss both direction and magnitude of any potential bias **Pages 10** |
| Interpretation | 20 | Give a cautious overall interpretation of results considering objectives, limitations, multiplicity of analyses, results from similar studies, and other relevant evidence **Pages 9-10** |
| Generalisability | 21 | Discuss the generalisability (external validity) of the study results **Pages 10** |
| Other information | | |
| Funding | 22 | Give the source of funding and the role of the funders for the present study and, if applicable, for the original study on which the present article is based **Page 10** |

*Give information separately for exposed and unexposed groups.

**Note:** An Explanation and Elaboration article discusses each checklist item and gives methodological background and published examples of transparent reporting. The STROBE checklist is best used in conjunction with this article (freely available on the Web sites of PLoS Medicine at http://www.plosmedicine.org/, Annals of Internal Medicine at http://www.annals.org/, and Epidemiology at http://www.epidem.com/). Information on the STROBE Initiative is available at www.strobe-statement.org.

**Fig S1.** Frequency of OGDS use in 2019. Vertical line=median among OGDS users. (n=1911)


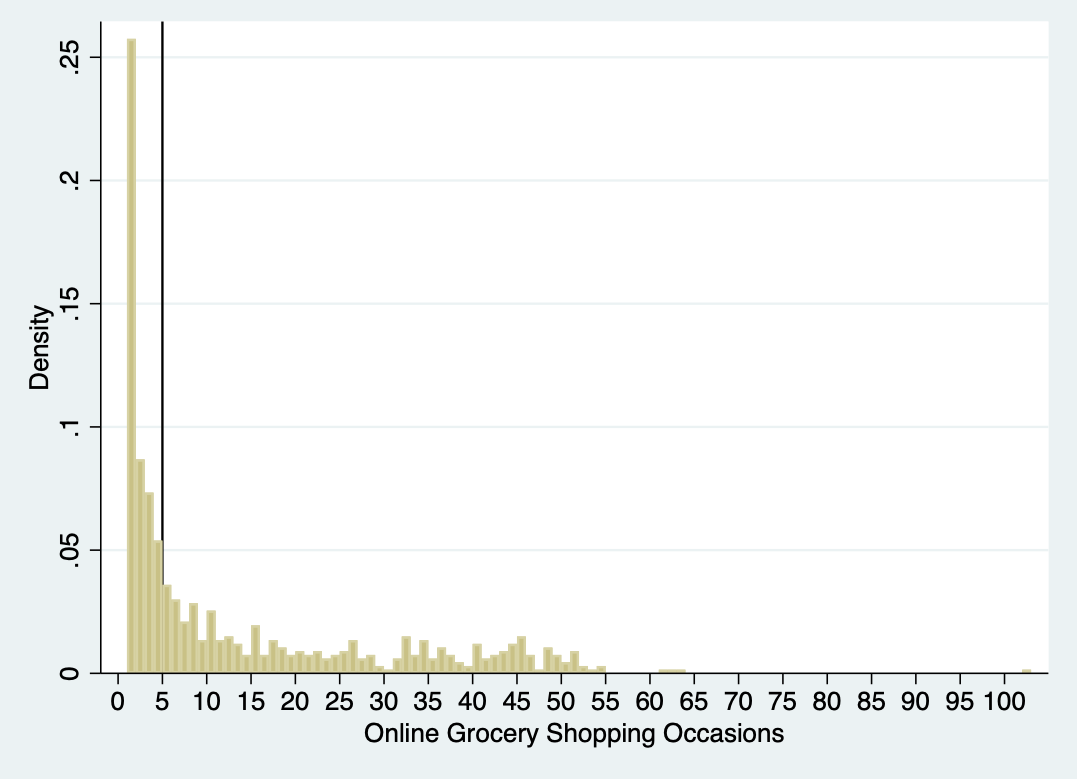


**Table S1.** Description of food groups

| **Food group** | **Examples** |
| --- | --- |
| Breads products - healthy | Breads, pizza bases, tortilla, pastry |
| Breads products - less healthy |  |
| Breakfast cereals - healthy | Breakfast cereals |
| Breakfast cereals - less healthy |  |
| Other morning goods | Pastries, muffins, scones, teacakes, waffles |
| Carbohydrates | Pasta, rice, other grains, potatoes, noodles, flour |
| Cheese - healthy | Cheese |
| Cheese - less healthy |  |
| Other dairy - healthy | Cream, yoghurt, fromage frais |
| Other dairy - less healthy |  |
| Proteins | Eggs, fish, poultry, seafood |
| Red meat - healthy | Beef, lamb, pork, offal |
| Red meat - unhealthy |  |
| Processed fish and meat - healthy | Canned and prepared fish and meat e.g. sausages, bacon, burgers, pate |
| Processed fish and meat - less healthy |  |
| Ready meals and convenience food - healthy | Prepared convenience foods (e.g. soups, salads), stuffed pasta, pizza, ready meals, sandwich fillers, pastes |
| Ready meals and convenience food - less healthy |  |
| Sauces and condiments - healthy | Cooking sauces, table sauces, salad dressing, vinegar, gravy, stock |
| Sauces and condiments - less healthy |  |
| Fresh fruits | Fresh fruit including prepared and frozen |
| Tinned fruits | Tinned and dried fruits |
| Vegetables | Vegetables including canned and jars (e.g. pickles), excluding legumes and potatoes |
| Legumes, nuts and seeds | Including beans and peas |
| Fat and oil | Cooking fats and oils |
| Savoury snacks | Including crisps, popcorn, savoury biscuits and olives |
| Chocolates and confectionery | Chocolate, sweets, sweet spreads (e.g. jam) |
| Puddings and biscuits | Biscuits, cereal and fruit bars, cakes, pastries, puddings, ice cream, custard, jelly |
| Milk - reduced-fat | Milk including non-dairy (e.g. soya, rice) |
| Milk - high-fat |  |
| Milk-based drinks | Milkshakes, yoghurt drinks, breakfast drinks |
| Non-milk-based drinks - healthy | Carbonated drinks, flavoured water, tea, coffee, mineral water |
| Non-milk-based drinks - less healthy |  |
| Juices | Pure juice and juice drinks |
| Alcohol | All alcoholic drinks |
| Others | Sugar, honey, syrup, sweeteners, table salt, slimming products |
| Food groups stratified by ‘healthy’ and ‘less healthy’ were determined by the UK’s Nutrient Profiling Model. | |

**Table S2.** Incidence Rate Ratio (95% CI) and predicted difference in adjusted weekly mean (95% CI) purchases of energy, nutrients, items per person and expenditure on groceries per person between households with above and below median OGDS use, stratified by food and drink (n=1911)

|  |  | **Incidence Rate Ratio (95% CI)** | **Predicted outcome for above median OGDS use, mean (95% CI) (n=353)** | **Predicted outcome for below median OGDS use, mean (95% CI) (n=1558)** | **Predicted difference, mean (95% CI)** |
| --- | --- | --- | --- | --- | --- |
| Food | Energy (kcal) | 1.14 (1.14 to 1.14) | 10,741 (10,729 to 10,751) | 9409 (9404 to 9,414) | **1331 (1318 to 1343)** |
|  | Fat (g) | 1.15 (1.14 to 1.15) | 488.6 (486.2 to 491.0) | 425.5 (424.5 to 426.5) | **63.1 (60.5 to 66.8)** |
|  | Saturated fat (g) | 1.17 (1.16 to 1.19) | 186.3 (184.8 to 187.8) | 158.5 (157.9 to 159.2) | **27.7 (26.1 to 29.4)** |
|  | Sugar (g) | 1.12 (1.12 to 1.13) | 430.8 (428.5 to 433.0) | 383.1 (382.1 to 384.1) | **47.7 (45.2 to 50.2)** |
|  | Salt (g) | 1.18 (1.16 to 1.21) | 38.5 (37.8 to 39.2) | 32.5 (32.3 to 32.8) | **6.0 (5.2 to 6.7)** |
|  | Items (n) | 1.17 (1.13 to 1.21) | 13.9 (13.5 to 14.3) | 11.9 (11.7 to 12.1) | **2.0 (1.6 to 2.5)** |
|  | Expenditure (£) | 1.25 (1.22 to 1.29) | 19.7 (19.2 to 20.2) | 15.7 (15.5 to 15.9) | **4.0 (3.5 to 4.5)** |
|  | Fat per 100 kcal | 1.00 (0.95 to 1.06) | 4.5 (4.3 to 4.7) | 4.5 (4.4 to 4.6) | 0.0 (-0.2 to 0.3) |
|  | Saturated fat per 100 kcal | 1.03 (0.94 to 1.13) | 1.7 (1.6 to 1.8) | 1.7 (1.6 to 1.7) | 0.0 (-0.1 to 0.2) |
|  | Sugar per 100 kcal | 0.98 (0.93 to 1.04) | 4.0 (3.8 to 4.2) | 4.0 (3.9 to 4.1) | -0.1 (-0.3 to 0.2) |
|  | Salt per 100 kcal | 1.04 (0.85 to 1.26) | 0.4 (0.3 to 0.4) | 0.3 (0.3 to 0.4) | 0.0 (-0.1 to 0.8) |
|  | Expenditure per 100 kcal | 1.11 (0.84 to 1.46) | 0.2 (0.1 to 0.2) | 0.2 (0.1 to 0.2) | 0.0 (-0.0 to 0.1) |
| Drink | Energy (kcal) | 1.12 (1.12 to 1.13) | 1184 (1180 to 1188) | 1055 (1053 to 1057) | **129 (125 to 133)** |
|  | Fat (g) | 1.08 (1.05 to 1.11) | 23.7 (23.1 to 24.2) | 21.9 (21.7 to 22.2) | **1.7 (1.2 to 2.3)** |
|  | Saturated fat (g) | 1.06 (1.02 to 1.09) | 14.1 (13.7 to 14.5) | 13.3 (13.2 to 13.5) | **0.7 (0.3 to 1.2)** |
|  | Sugar (g) | 1.17 (1.15 to 1.18) | 113.8 (112.6 to 115.0) | 97.5 (97.1 to 98.0) | **16.3 (15.0 to 17.6)** |
|  | Salt (g) | 1.10 (1.01 to 1.21) | 1.8 (1.7 to 2.0) | 1.7 (1.6 to 1.7) | **0.2 (0.0 to 0.3)** |
|  | Items (n) | 1.23 (1.14 to 1.33) | 2.7 (2.5 to 2.8) | 2.2 (2.1 to 2.2) | **0.5 (0.3 to 0.7)** |
|  | Expenditure (£) | 1.23 (1.16 to 1.29) | 5.4 (5.2 to 5.7) | 4,4 (4.3 to 4.5) | **1.0 (0.7 to 1.3)** |
|  | Fat per 100 kcal | 0.99 (0.91 to 1.07) | 2.2 (2.0 to 2.3) | 2.2 (2.1 to 2.3) | -0.0 (-0.2 to 0.1) |
|  | Saturated fat per 100 kcal) | 0.95 (0.85 to 1.05) | 1.3 (1.1 to 1.4) | 1.3 (1.3 to 1.4) | -0.1 (-0.2 to 0.1) |
|  | Sugar per 100 kcal | 1.01 (0.97 to 1.05) | 10.3 (9.9 to 10.6) | 10.1 (10.0 to 10.3) | 0.1 (-0.3 to 0.5) |
|  | Salt per 100 kcal | 1.01 (0.76 to 1.34) | 0.2 (0.1 to 0.2) | 0.2 (0.2 to 0.2) | 0.0 (-0.0 to 0.1) |
|  | Expenditure per 100 kcal | 1.11 (0.93 to 1.32) | 0.5 (0.4 to 0.5) | 0.4 (0.4 to 0.5) | 0.0 (-0.0 to 0.1) |
| Poisson model adjusted for sex and age of the main food shopper, occupational social class, household income, proportion of household members that were children, and region.  **Bold**, significant at 95% confidence level. | | | | | |

**Table S3.** Incidence Rate Ratio (95% CI) and predicted difference in adjusted mean (95% CI) energy purchased (kcal) per person per week, stratified by healthy (non-HFSS) and less healthy (HFSS) food groups (n=1911)

|  |  | **Incidence Rate Ratio (95% CI)** | **Predicted outcome for above median OGDS use, mean (95% CI) (n=353)** | **Predicted outcome for below median OGDS use, mean (95% CI) (n=1558)** | **Predicted difference, mean (95% CI)** |
| --- | --- | --- | --- | --- | --- |
| Healthy | Breads products – healthy | 1.11 (1.03 to 1.20) | 985 (915 to 1055) | 885 (854 to 915) | **100 (24 to 176)** |
|  | Breakfast cereals - healthy | 1.03 (0.91 to 1.17) | 304 (270 to 337) | 294 (277 to 310) | 10 (-27 to 47) |
|  | Other morning goods | 1.12 (0.96 to 1.31) | 138 (119 to 158) | 123 (115 to 131) | 15 (-6 to 36) |
|  | Pasta, rice and other grains | 1.10 (1.01 to 1.21) | 656 (601 to 712) | 594 (571 to 618) | **62 (2 to 122)** |
|  | Cheese – healthy | 0.74 (0.52 to 1.06) | 4 (3 to 6) | 6 (5 to 7) | -2 (-3 to 0) |
|  | Other dairy – healthy | 1.20 (1.03 to 1.40) | 111 (96 to 126) | 92 (86 to 98) | **19 (2 to 35)** |
|  | Proteins | 1.13 (1.02 to 1.24) | 577 (527 to 626) | 512 (489 to 535) | **65 (10 to 119)** |
|  | Red meat – healthy | 1.11 (0.98 to 1.26) | 153 (136 to 171) | 138 (130 to 147) | 15 (-4 to 34) |
|  | Processed fish and meat – healthy | 1.25 (1.08 to 1.45) | 206 (178 to 234) | 165 (156 to 173) | **41 (12 to 71)** |
|  | Ready meals and convenience food – healthy | 1.22 (1.11 to 1.33) | 713 (655 to 771) | 585 (562 to 608) | **128 (65 to 190)** |
|  | Sauces and condiments – healthy | 1.09 (0.95 to 1.25) | 44 (39 to 50) | 40.5 (38.1 to 43.0) | 4 (-2 to 10) |
|  | Fresh fruits | 0.99 (0.88 to 1.11) | 165 (148 to 183) | 167 (159 to 176) | -2 (-21 to 17) |
|  | Tinned fruits | 0.99 (0.75 to 1.29) | 6 (5 to 8) | 6 (5 to 7) | -0 (-2 to 2) |
|  | Vegetables, excl. legumes and potatoes | 1.11 (1.02 to 1.21) | 323 (299 to 348) | 291 (280 to 302) | **32 (5 to 59)** |
|  | Legumes, nuts and seeds | 1.02 (0.91 to 1.16) | 190 (168 to 211) | 185 (176 to 195) | **5 (19 to 28)** |
|  | Milk - reduced-fat | 1.14 (1.01 to 1.28) | 428 (383 to 473) | 376 (358 to 395) | **52 (3 to 100)** |
|  | Milk-based drinks | 0.85 (0.65 to 1.13) | 26 (19 to 33) | 31 (27 to 34) | -5 (-12 to 3) |
|  | Non-milk-based drinks – healthy | 1.28 (1.10 to 1.49) | 46 (40 to 51) | 36 (33 to 38) | **10 (4 to 16)** |
|  | Juices | 1.23 (1.08 to 1.41) | 124 (109 to 138) | 100 (94 to 107) | **23 (7 to 39)** |
| Less healthy | Breads products - less healthy | 0.99 (0.88 to 1.12) | 161 (143 to 179) | 162 (153 to 171) | -1 (-21 to 19) |
|  | Breakfast cereals - less healthy | 1.03 (0.87 to 1.21) | 162 (138 to 186) | 158 (145 to 171) | 4 (-23 to 31) |
|  | Cheese - less healthy | 1.29 (1.18 to 1.41) | 684 (629 to 739) | 531 (509 to 553) | **153 (94 to 212)** |
|  | Other dairy - less healthy | 1.13 (0.96 to 1.32) | 157 (135 to 178) | 139 (129 to 149) | 18 (-6 to 44) |
|  | Red meat – unhealthy | 1.10 (0.92 to 1.32) | 96 (82 to 110) | 87 (77 to 97) | 9 (-8 to 26) |
|  | Processed fish and meat - less healthy | 1.30 (1.18 to 1.44) | 702 (637 to 767) | 540 (518 to 562) | **162 (94 to 231)** |
|  | Ready meals and convenience food - less healthy | 1.14 (1.02 to 1.28) | 311 (279 to 343) | 272 (257 to 287) | **39 (4 to 74)** |
|  | Sauces and condiments - less healthy | 1.21 (1.09 to 1.33) | 223 (204 to 243) | 185 (177 to 194) | **38 (17 to 60)** |
|  | Fat and oil | 1.09 (1.00 to 1.20) | 872 (798 to 946) | 798 (766 to 831) | 74 (-6 to 154) |
|  | Savoury snacks | 1.12 (1.01 to 1.25) | 512 (461 to 564) | 456 (435 to 478) | 56 (1 to 111) |
|  | Chocolates and confectionery | 1.11 (0.99 to 1.24) | 632 (565 to 699) | 571 (546 to 596) | 62 (-10 to 133) |
|  | Puddings and biscuits | 1.16 (1.07 to 1.26) | 1394 (1288 to 1501) | 1203 (1160 to 1246) | **191 (77 to 305)** |
|  | Milk - high-fat | 0.91 (0.70 to 1.18) | 125 (96 to 155) | 137 (121 to 154) | -12 (-45 to 21) |
|  | Non-milk-based - less healthy | 1.38 (1.09 to 1.73) | 109 (85 to 132) | 79 (73 to 85) | **30 (5 to 54)** |
|  | Alcohol | 1.11 (0.91 to 1.35) | 328 (270 to 386) | 296 (268 to 324) | 32 (-32 to 96) |
|  | Others | 1.18 (0.99 to 1.40) | 261 (221 to 302) | 222 (207 to 238) | 39 (-4 to 83) |
| Poisson model adjusted for sex and age of the main food shopper, occupational social class, household income, proportion of household members that were children, and region.  OGDS = Online grocery delivery service | | | | | |

**Table S4.** Incidence Rate Ratio (95% CI) and predicted difference in adjusted mean (95% CI) proportion of energy purchased from food groups for online and in-store purchases (n=665)

|  | **Incidence Rate Ratio (95% CI)** | **Predicted outcome for online shops, mean (95% CI)** | **Predicted outcome for in-store shops, mean (95% CI)** | **Predicted difference, mean (95% CI)** |
| --- | --- | --- | --- | --- |
| Breads products - healthy | -0.46 (-0.56 to -0.36) | 5 (5 to 6) | 8 (8 to 9) | **-3 (-4 to -3)** |
| Breads products - less healthy | -0.60 (-0.75 to -0.45) | 1 (1 to 1) | 2 (1 to 2) | **-1 (-1 to -1)** |
| Breakfast cereals - healthy | 0.15 (-0.04 to 0.33) | 3 (2 to 3) | 2 (2 to 3) | 0 (-0 to 1) |
| Breakfast cereals - less healthy | -0.10 (-0.27 to 0.07) | 1 (1 to 2) | 1 (1 to 2) | -0 (-0 to 0) |
| Other morning goods | -0.61 (-0.86 to -0.35) | 1 (1 to 1) | 1 (1 to 2) | **-1 (-1 to -0)** |
| Pasta, rice and other grains | -0.03 (-0.13 to 0.08) | 6 (5 to 6) | 6 (5 to 6) | -0 (-1 to 0) |
| Cheese - healthy | -0.33 (-0.73 to 0.07) | 0 (0 to 0) | 0 (0 to 0) | -0 (-0 to 0) |
| Cheese - less healthy | -0.23 (-0.33 to -0.13) | 5 (4 to 5) | 6 (6 to 6) | **-1 (-2 to -1)** |
| Other dairy - healthy | -0.04 (-0.17 to 0.09) | 1 (1 to 1) | 1 (1 to 1) | -0 (-0 to 0) |
| Other dairy - less healthy | -0.40 (-0.63 to -0.17) | 1 (1 to 1) | 1 (1 to 2) | **-1 (-1 to -0)** |
| Proteins (egg, fish, white meat, meat substitutes) | -0.12 (-0.25 to 0.00) | 4 (4 to 5) | 5 (5 to 5) | **-1 (-1 to -0)** |
| Red meat - healthy | -0.02 (-0.28 to 0.23) | 1 (1 to 2) | 1 (1 to 2) | -0 (-0 to 0) |
| Red meat - unhealthy | -0.10 (-0.31 to 0.11) | 1 (1 to 1) | 1 (1 to 1) | -0 (-0 to 0) |
| Processed fish and meat - healthy | -0.17 (-0.38 to 0.05) | 2 (1 to 2) | 2 (2 to 2) | -0 (-1 to 0) |
| Processed fish and meat - less healthy | -0.29 (-0.40 to -0.19) | 4 (4 to 5) | 6 (5 to 6) | **-1 (-2 to -1)** |
| Ready meals and convenience food - healthy | -0.23 (-0.32 to -0.14) | 5 (4 to 5) | 6 (6 to 6) | **-1 (-2 to -1)** |
| Ready meals and convenience food - less healthy | -0.29 (-0.52 to -0.06) | 2 (2 to 3) | 3 (3 to 3) | **-1 (-1 to -0)** |
| Sauces and condiments - healthy | -0.04 (-0.29 to 0.21) | 0 (0 to 1) | 0 (0 to 1) | -0 (-0 to 0) |
| Sauces and condiments - less healthy | 0.06 (-0.16 to 0.29) | 2 (2 to 2) | 2 (2 to 2) | 0 (-0 to 1) |
| Fresh fruits | 0.14 (-0.19 to 0.47) | 2 (1 to 3) | 2 (2 to 2) | 0 (-0 to 1) |
| Tinned fruits | -0.09 (-0.43 to 0.24) | 0 (0 to 0) | 0 (0 to 0) | -0 (-0 to 0) |
| Vegetables, excl. legumes and potatoes | 0.31 (0.10 to 0.52) | 4 (3 to 5) | 3 (3 to 3) | **1 (0 to 2)** |
| Legumes, nuts and seeds | 0.04 (-0.10 to 0.17) | 2 (2 to 2) | 2 (2 to 2) | 0 (-0 to 0) |
| Fat and oil | -0.14 (-0.28 to -0.01) | 6 (5 to 7) | 7 (6 to 7) | **-1 (-2 to -0)** |
| Savoury snacks | -0.19 (-0.34 to -0.04) | 4 (3 to 4) | 5 (4 to 5) | **-1 (-1 to -0)** |
| Chocolates and confectionery | -0.31 (-0.49 to -0.13) | 4 (3 to 5) | 6 (5 to 6) | **-2 (-2 to -1)** |
| Puddings and biscuits | -0.34 (-0.44 to -0.24) | 8 (7 to 9) | 11 (11 to 12) | **-3 (-4 to -3)** |
| Milk - reduced-fat | -0.31 (-0.42 to -0.20) | 3 (2 to 3) | 3 (3 to 4) | **-1 (-1 to -1)** |
| Milk - high-fat | -0.43 (-0.66 to -0.20) | 1 (1 to 1) | 1 (1 to 1) | **-0 (-1 to -0)** |
| Milk-based drinks | -0.27 (-0.57 to 0.04) | 0 (0 to 0) | 0 (0 to 0) | -0 (-0 to 0) |
| Non-milk-based - healthy | 1.73 (1.25 to 2.20) | 2 (1 to 3) | 0 (0 to 0) | **2 (1 to 2)** |
| Non-milk-based - less healthy | 0.35 (-0.07 to 0.76) | 1 (1 to 2) | 1 (1 to 1) | 0 (-0 to 1) |
| Juices | 0.22 (-0.09 to 0.54) | 1 (1 to 2) | 1 (1 to 1) | 0 (-0 to 1) |
| Alcohol | 0.40 (0.15 to 0.65) | 4 (3 to 5) | 3 (2 to 3) | **1 (0 to 2)** |
| Others (sugar, table salt, slimming products) | 0.15 (-0.11 to 0.41) | 2 (2 to 3) | 2 (2 to 2) | 0 (-0 to 1) |
| Poisson model adjusted for sex and age of the main food shopper, occupational social class, household income, proportion of household members that were children, and region.  **Bold**, significant at 95% confidence level. | | | | |

**Table S5.** Incidence Rate Ratio (95% CI) and predicted difference in adjusted weekly mean (95% CI) purchases of energy, nutrients, items per person and expenditure on groceries per person between regular OGDS users and non-regular OGDS users (n=1911)

|  | **Incidence Rate Ratio (95% CI)** | **Predicted outcome for regular online users, mean (95% CI) (n=147)** | **Predicted outcome for non-regular users, mean (95% CI) (n=1764)** | **Predicted difference, mean (95% CI)** |
| --- | --- | --- | --- | --- |
| Energy (kcal) | 1.21 (1.21 to 1.21) | 12,772 (12,753 to 12,792) | 10,565 (10,560 to 10,569) | 2208 (2188 to 2228) |
| Fat (g) | 1.20 (1.19 to 1.21) | 543.6 (539.6 to 547.7) | 452.3 (451.3 to 453.3) | 91.4 (87.2 to 95.5) |
| Saturated fat (g) | 1.22 (1.21 to 1.24) | 212.5 (210.0 to 215.1) | 174.1 (173.5 to 174.7) | 38.4 (35.8 to 41.0) |
| Sugar (g) | 1.20 (1.19 to 1.21) | 580.4 (576.2 to 584.6) | 485.1 (484.0 to 486.1) | 95.3 (91.0 to 99.7) |
| Salt (g) | 1.22 (1.18 to 1.25) | 42.2 (41.1 to 43.4) | 34.7 (34.5 to 35.0) | 7.5 (6.4 to 8.7) |
| Items (n) | 1.24 (1.19 to 1.29) | 20.0 (19.2 to 20.7) | 16.1 (15.9 to 16.3) | 3.9 (3.1 to 4.7) |
| Expenditure | 1.30 (1.26 to 1.35) | 31.5 (30.6 to 32.5) | 24.2 (24.0 to 24.4) | 7.3 (6.3 to 8.3) |
| Fat per 100 kcal | 0.98 (0.91 to 1.07) | 4.2 (3.9 to 4.5) | 4.3 (4.2 to 4.4) | -0.1 (-0.4 to 0.3) |
| Saturated fat per 100 kcal | 1.00 (0.88 to 1.15) | 1.6 (1.4 to 1.8) | 1.6 (1.6 to 1.7) | 0.0 (-0.2 to 0.2) |
| Sugar per 100 kcal | 0.99 (0.92 to 1.08) | 4.5 (4.2 to 4.9) | 4.6 (4.5 to 4.7) | -0.0 (-0.4 to 0.3) |
| Salt per 100 kcal | 1.01 (0.75 to 1.36) | 0.3 (0.3 to 0.4) | 0.3 (0.3 to 0.4) | 0.0 (-0.1 to 0.1) |
| Expenditure per 100 kcal | 1.12 (0.80 to 1.57) | 0.3 (0.2 to 0.3) | 0.2 (0.2 to 0.3) | 0.0 (-0.1 to 0.1) |
| Poisson model adjusted for sex and age of the main food shopper, occupational social class, household income, proportion of household members that were children, and region. | | | | |

**Table S6.** Coefficient (95% CI) and predicted difference in adjusted weekly mean (95% CI) energy, nutrients, items from HFSS purchases as a proportion of all grocery food and drink purchases and expenditure on HFSS products as a proportion of all food and drink grocery expenditure between regular OGDS users and non-regular OGDS users (n=1911)

|  | **Coef. (95% CI)** | **Predicted outcome for regular online users, mean (95% CI) (n=147)** | **Predicted outcome for non-regular users, mean (95% CI) (n=1764)** | **Predicted difference, mean (95% CI)** |
| --- | --- | --- | --- | --- |
| Energy (kcal) | 0.01 (-0.01 to 0.02) | 52.6 (51.1 to 54.1) | 52.5 (52.1 to 53.0) | 0.1 (-1.5 to 1.6) |
| Fat (g) | 0.01 (-0.01 to 0.02) | 71.4 (69.9 to 72.8) | 70.7 (70.3 to 71.1) | 0.7 (-0.8 to 0.2) |
| Saturated fat (g) | 0.00 (-0.01 to 0.02) | 76.3 (74.9 to 77.7) | 76.1 (75.7 to 76.6) | 0.2 (-1.3 to 1.6) |
| Sugar (g) | -0.02 (-0.04 to 0.00) | 54.8 (52.6 to 56.9) | 56.8 (56.2 to 57.4) | -2.0 (-4.3 to 0.3) |
| Salt (g) | 0.00 (-0.01 to 0.02) | 64.3 (62.5 to 66.0) | 63.8 (63.3 to 64.3) | 0.4 (-1.3 to 2.2) |
| Items (n) | -0.00 (-0.02 to 0.01) | 37.3 (35.8 to 38.8) | 37.7 (37.3 to 38.1) | -0.4 (-2.0 to 0.1) |
| Expenditure (£) | 0.00 (-0.02 to 0.02) | 39.7 (38.2 to 41.3) | 40.0 (39.2 to 40.1) | 0.1 (-1.6 to 1.7) |
| Poisson model adjusted for sex and age of the main food shopper, occupational social class, household income, proportion of household members that were children, and region. | | | | |

**Table S7.** Incidence Rate Ratio (95% CI) and predicted difference in adjusted weekly mean (95% CI) purchases of energy, nutrients, items per person and expenditure on groceries per person between OGDS users and non-OGDS users (n=1911)

|  | **Incidence Rate Ratio (95% CI)** | **Predicted outcome for OGDS users, mean (95% CI) (n=668)** | **Predicted outcome for non-OGDS, users, mean (95% CI) (n=1,243)** | **Predicted difference, mean (95% CI)** |
| --- | --- | --- | --- | --- |
| Energy (kcal) | 1.07 (1.07 to 1.07) | 11,224 (11,216 to 11,232) | 10,468 (10,462 to 10,473) | **756 (746 to 766)** |
| Fat (g) | 1.07 (1.07 to 1.08) | 481.4 (479.6 to 483.1) | 447.4 (446.2 to 448.5) | **34.0 (31.9 to 36.1)** |
| Saturated fat (g) | 1.09 (1.08 to 1.10) | 187.1 (186.1 to 188.2) | 171.7 (171.0 to 172.4) | **15.4 (14.1 to 16.7)** |
| Sugar (g) | 1.07 (1.07 to 1.08) | 514.7 (512.9 to 516.5) | 480.3 (479.1 to 481.6) | **34.3 (32.1 to 36.5)** |
| Salt (g) | 1.10 (1.08 to 1.12) | 37.5 (37.0 to 38.0) | 34.1 (33.8 to 34.5) | **3.4 (2.8 to 4.0)** |
| Items (n) | 1.12 (1.09 to 1.15) | 17.6 (17.3 to 18.0) | 15.7 (15.5 to 15.9) | **1.9 (1.5 to 2.3)** |
| Expenditure (£) | 1.18 (1.16 to 1.20) | 27.5 (27.1 to 27.9) | 23.3 (23.0 to 23.6) | **4.2 (3.7 to 4.7)** |
| Fat per 100 kcal | 1.00 (0.96 to 1.05) | 4.3 (4.1 to 4.4) | 4.3 (4.1 to 4.4) | 0.0 (-0.2 to 0.2) |
| Saturated fat per 100 kcal | 1.02 (0.94 to 1.10) | 1.7 (1.6 to 1.7) | 1.6 (1.6 to 1.7) | 0.0 (-0.1 to 0.1) |
| Sugar per 100 kcal | 0.98 (0.94 to 1.03) | 4.5 (4.4 to 4.7) | 4.6 (4.5 to 4.7) | -0.1 (-0.3 to 0.1) |
| Salt per 100 kcal | 1.02 (0.87 to 1.21) | 0.3 (0.3 to 0.4) | 0.3 (0.3 to 0.4) | 0.0 (-0.0 to 0.1) |
| Expenditure per 100 kcal | 1.12 (0.92 to 1.36) | 0.3 (0.2 to 0.3) | 0.2 (0.2 to 0.2) | 0.0 (-0.0 to 0.1) |
| Poisson model adjusted for sex and age of the main food shopper, occupational social class, household income, proportion of household members that were children, and region. | | | | |

**Table S8.** Coefficient (95% CI) and predicted difference in adjusted weekly mean (95% CI) energy, nutrients, items from HFSS purchases as a proportion of all grocery food and drink purchases and expenditure on HFSS products as a proportion of all food and drink grocery expenditure between regular OGDS users and non-regular OGDS users (n=1911)

|  | **Coef. (95% CI)** | **Predicted outcome for OGDS users, mean (95% CI) (n=668)** | **Predicted outcome for non-OGDS users, mean (95% CI) (n=1243)** | **Predicted difference, mean (95% CI)** |
| --- | --- | --- | --- | --- |
| Energy (kcal) | 0.01 (0.00 to 0.02) | 53.1 (52.4 to 53.8) | 52.2 (51.7 to 52.7) | **0.9 (0.0 to 1.8)** |
| Fat (g) | 0.01 (0.00 to 0.02) | 71.3 (70.7 to 72.0) | 70.4 (70.0 to 70.9) | **0.9 (0.1 to 1.7)** |
| Saturated fat (g) | 0.01 (0.01 to 0.02) | 77.1 (76.4 to 77.7) | 75.7 (75.2 to 76.2) | **1.4 (0.6 to 2.2)** |
| Sugar (g) | 0.01 (-0.01 to 0.02) | 57.1 (56.0 to 58.1) | 56.4 (55.6 to 57.1) | 0.7 (-0.6 to 1.9) |
| Salt (g) | 0.01 (-0.00 to 0.02) | 64.4 (63.6 to 65.2) | 63.6 (63.0 to 64.1) | 0.9 (-0.1 to 1.9) |
| Items (n) | 0.01 (-0.00 to 0.02) | 38.2 (37.5 to 38.9) | 37.4 (36.9 to 37.9) | 0.7 (-0.1 to 1.6) |
| Expenditure (£) | 0.00 (-0.01 to 0.01) | 39.9 (39.2 to 40.7) | 39.5 (39.0 to 40.1) | 0.4 (-0.5 to 1.3) |
| Poisson model adjusted for sex and age of the main food shopper, occupational social class, household income, proportion of household members that were children, and region. | | | | |

**Table S9.** Incidence Rate Ratio (95% CI) and predicted difference in adjusted weekly mean (95% CI) purchases of energy, nutrients, items per person and expenditure on groceries per person between households with above and below median OGDS use

additionally adjusted for BMI (n=1544)

|  | **Incidence Rate Ratio (95% CI)** | **Predicted outcome for above median OGDS use, mean (95% CI) (n=1,272)** | **Predicted outcome for below median OGDS use, mean (95% CI) (n=272)** | **Predicted difference, mean (95% CI)** |
| --- | --- | --- | --- | --- |
| Energy (kcal) | 1.12 (1.12 to 1.12) | 11,978 (11,964 to 11,991) | 10,709 (10,703 to 10,715) | **1269 (1254 to 1284)** |
| Fat (g) | 1.12 (1.12 to 1.13) | 514.5 (511.7 to 517.4) | 457.7 (456.5 to 458.9) | **56.9 (53.8 to 60.0)** |
| Saturated fat (g) | 1.15 (1.14 to 1.17) | 203.3 (201.5 to 205.1) | 176.1 (175.4 to 176.8) | **27.2 (25.3 to 29.1)** |
| Sugar (g) | 1.13 (1.12 to 1.13) | 554.4 (551.4 to 557.3) | 492.1 (490.8 to 493.3) | **62.3 (59.1 to 65.5)** |
| Salt (g) | 1.15 (1.12 to 1.17) | 40.1 (39.3 to 40.9) | 35.0 (34.7 to 35.3) | **5.1 (4.3 to 6.0)** |
| Items (n) | 1.16 (1.12 to 1.19) | 18.8 (18.3 to 19.3) | 16.3 (16.1 to 16.5) | **2.5 (1.9 to 3.1)** |
| Expenditure (£) | 1.24 (1.21 to 1.27) | 30.1 (29.4 to 30.8) | 24.3 (24.0 to 24.6) | **5.8 (5.1 to 6.5)** |
| Poisson model adjusted for sex and age of the main food shopper, occupational social class, household income, proportion of household members that were children, region, and BMI. | | | | |

**Supplementary Table S10.** Coefficient (95% CI) and predicted difference in adjusted weekly mean (95% CI) energy, nutrients, items from HFSS purchases as a proportion of all grocery food and drink purchases and expenditure on HFSS products as a proportion of all food and drink grocery expenditure between households with above and below median OGDS use additionally adjusted for BMI (n=1544)

|  | **Coef. (95% CI)** | **Predicted outcome for above median OGDS use, mean (95% CI) (n=1272)** | **Predicted outcome for below median OGDS use, mean (95% CI) (n=272)** | **Predicted difference, mean (95% CI)** |
| --- | --- | --- | --- | --- |
| Energy (kcal) | 0.00 (-0.01 to 0.01) | 52.6 (51.5 to 53.7) | 52.4 (51.9 to 52.9) | 1.9 (-1.0 to 1.4) |
| Fat (g) | 0.01 (-0.01 to 0.02) | 71.2 (70.1 to 72.2) | 70.5 (70.0 to 71.0) | 0.6 (-0.5 to 1.8) |
| Saturated fat (g) | 0.01 (-0.0 to 0.02) | 77.0 (75.9 to 78.1) | 76.0 (75.5 to 76.4) | 1.0 (-0.1 to 2.2) |
| Sugar (g) | -0.00 (-0.02 to 0.02) | 56.6 (55.0 to 58.2) | 56.6 (55.9 to 57.3) | -0.0 (-1.8 to 1.8) |
| Salt (g) | 0.01 (-0.01 to 0.02) | 64.4 (63.2 to 65.7) | 63.6 (63.1 to 64.2) | 0.8 (-0.6 to 2.2) |
| Items (n) | 0.00 (-0.01 to 0.01) | 37.6 (36.5 to 38.7) | 37.4 (37.0 to 38.0) | 0.2 (-1.1 to 1.4) |
| Expenditure (£) | 0.00 (-0.01 to 0.01) | 39.4 (38.2 to 40.5) | 39.4 (38.8 to 39.9) | 0.0 (-1.3 to 1.3) |
| Poisson model adjusted for sex and age of the main food shopper, occupational social class, household income, proportion of household members that were children, region, and BMI. | | | | |
